# Time delays and margins in gated radiotherapy

**DOI:** 10.1120/jacmp.v10i3.2896

**Published:** 2009-07-28

**Authors:** Wendy L. Smith, Nathan Becker

**Affiliations:** ^1^ Department of Medical Physics Tom Baker Cancer Centre Calgary Alberta Canada T2N 4N2

**Keywords:** respiratory gating, time delay, margin, gated radiotherapy

## Abstract

In gated radiotherapy, the accuracy of treatment delivery is determined by the accuracy with which both the imaging and treatment beams are gated. Time delays are of four types: (1) beam on imaging time delay is the time between the target entering the gated region and the first gated image acquisition; (2) beam off imaging time delay is the time between the target exiting a gated region and the last image acquisition; (3) beam on treatment time delay is the time between the target entering the gated region and the treatment beam on; and (4) beam off treatment time delay is the time between the target exiting the gated region and treatment beam off. Asynchronous time delays for the imaging and treatment systems may increase the required internal target volume (ITV) margin. We measured time delay on three fluoroscopy systems, and three linear accelerator treatment beams, varying gating type (amplitude vs. phase), beam energy, dose rate, and period. The average beam on imaging time delays were −0.04±0.05sec (amplitude, 1 SD), −0.11±0.04sec (phase); while the average beam off imaging time delays were −0.18±0.08sec (amplitude) and −0.15±0.04sec (phase). The average beam on treatment time delays were +0.09±0.02sec (amplitude, 1 SD), +0.10±0.03sec (phase); while the average beam off time delays for treatment beams were +0.08±0.02sec (amplitude) and +0.07±0.02sec (phase). The negative value indicates the images were acquired early, and the positive values show the treatment beam was triggered late. We present a technique for calculating the margin necessary to account for time delays. We found that the difference between these imaging and treatment time delays required a significant increase in the ITV margin in the direction of tumor motion at the gated level.

PACS number: 87.53.Dq

## I. INTRODUCTION

**Figure 1:**
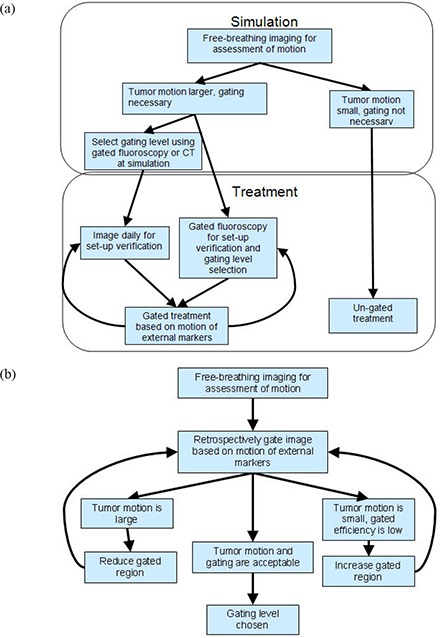
A workflow diagram describing the use of gated radiotherapy based on the motion of external surrogate markers (a). Gating levels may be chosen at simulation and may or may not be adjusted or verified daily. Selection of appropriate gating levels is shown in (b). Gating levels are assessed using imaging, then transferred to a treatment system for gated delivery. Even when the imaging beam and treatment beam are on the same machine, the mechanisms for gating them may differ.

Respiratory motion is a limiting factor in the accuracy of external beam treatment delivery for many sites in the thorax and abdomen. Respiration necessitates increased margins to accurately cover the clinical target volume (CTV), increasing the dose to normal tissue and limiting the dose deliverable to the target. One way to overcome these challenges is to use respiratory gating. Gating systems track the respiratory cycle and synchronize beam delivery with it. A flow diagram describing how gating fits into the larger context of a radiotherapy treatment is shown in Fig. 1.

Gating systems come in a variety of forms.^(^ ^1^ ^,^ ^2^ ^,^ ^3^ ^,^ ^4^ ^)^ One of the more widely used is the Varian Real‐time Position Management (RPM) system (Varian Medical Systems, Inc., Palo Alto, CA), which uses infrared (IR) markers placed on the patient's chest or abdomen (indicating displacement with respiration) as an external indicator of breathing motion to gate a treatment or imaging beam. This type of system assumes that the motion of external markers correlates with the tumor motion. Images of the moving target, or a fiducial representing the motion of that target, are also gated in order to assess the correlation between the target and external surrogate, as recommended by the AAPM's TG‐76.^(1)^ Gating may be accomplished based on the phase of the breathing cycle or based on the position (or amplitude of motion) of the markers in space. The relative merits of these two techniques are discussed in the literature.^(^ ^2^ ^,^ ^5^ ^)^

The most basic parameter influencing beam delivery accuracy is the time between when the markers enter or leave the specified position range for amplitude‐based gating or phase for phase‐based gating, and when an image is obtained or linear accelerator beam starts or ceases delivery.^(6)^ This is the *time delay* of the gated beam, and is seen in both prospectively (where a beam is turned on or off in real time by the gating system) and retrospectively (where a series of images are analyzed based on the time at which they were obtained to simulate a gated moving image or 4DCT) gated systems. The beam start delay and beam stop delay may not be identical, and are measured separately in this work. Manufacturers may quote the time delay for the software processing of the external marker tracking system,^(4)^ but this should not be confused with the total time delay of beam delivery, of which it is only a component.

In this paper, we discuss time delay measurements for RPM‐gated fluoroscopy on one conventional simulator and two on‐board imaging (OBI) systems (Varian Medical Systems, Inc., Palo Alto, CA), as well as for three RPM‐gated linear accelerator treatment beams. The types of measurements discussed and their possible implications for patient treatments can easily be applied to other gating systems.

## II. MATERIALS AND METHODS

### A. Gated imaging

**Figure 2:**
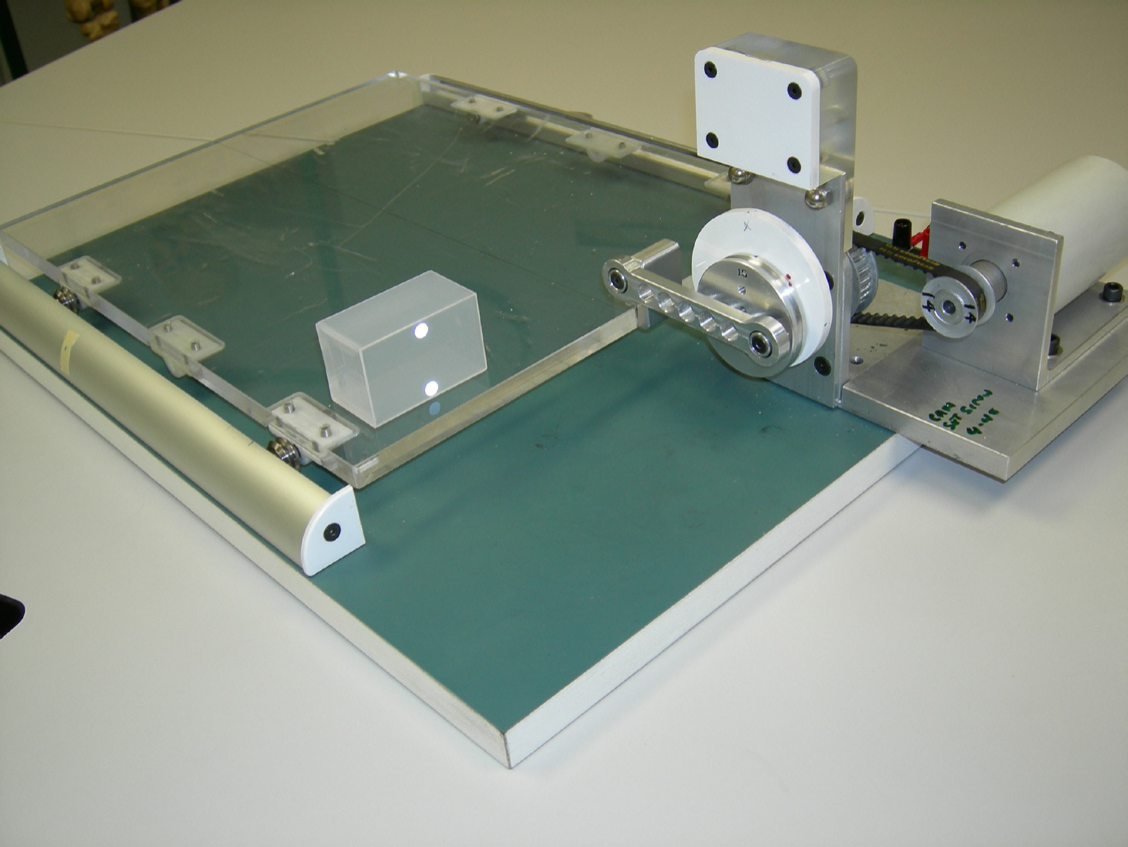
The motion phantom consists of a rotating disk attached to a sliding platform by a metal bar 8 cm in length. The platform moves in a modified sin wave. For the imaging time delay measurements, a metal marker was placed on the moving platform beside a static ruler. For treatment time delays, we placed Kodak PPL film and a 1.5 mm thick buildup layer of copper on the platform. The linear accelerator beam was collimated by a 4 mm cone placed on a static platform approximately 5 cm above the film. The IR camera monitored actual vertical motion (IR markers on cam‐device).

To measure time delay, we built a simple respiratory phantom consisting of a moving platform on guide rails driven by a shaft mounted on a disk as shown in Fig. 2. The radius at which the shaft was mounted and the speed of the motor could be varied, changing the frequency and amplitude of the motion. For time delay measurements, markers were mounted directly on the moving platform, so the markers moved in a modified sine wave.

**Figure 3:**
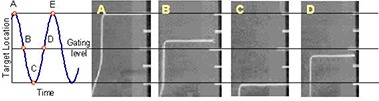
The method for measuring the time delay for fluoroscopy. The curve at left shows the phantom motion. Points A and E correspond to 100% amplitude and 0% phase; B: 50% amplitude and 25% phase; C: 0% amplitude and 50% phase; D: 50% amplitude and 75% phase. In a system with no time delays, the marker should be at the top horizontal line in image A, the middle line in images B and D and at the bottom line in C. In order to find the images corresponding to the times A, B, C, D, E, a series of gated playbacks were examined. For instance, when a gated region of 25% to 75% phase was chosen, the first image in the playback, representing time B, was the image labeled B above. Image C was found near the middle of the playback and the last image seen was image D. Because the marker is moving slowly or not at all around points A, C and E, the location of the marker in these images was relatively stable. At points B and D, however, the velocity of the marker could be easily calculated from the known phantom motion. Using this and the distance the marker was from its anticipated location, we calculated the time delay of the gated imaging. In this example, image B can be used to find the beam on time delay; image D to find the beam off time delay.

A radiographic marker was secured to the moving platform and a metal static scale was placed beside it so we could measure the position of the platform as a function of time on the gated images. The marker was set to a zero point at mid‐motion, (positions B and D in Fig. 3).

Fluoroscopic movies of the moving phantom, up to 45 seconds in length, were taken. A series of gated playbacks were examined. The gating level chosen was at mid‐motion (positions B and D in Fig. 3), where the marker velocity was high and acceleration small. In a system with no time delay, the image of the radiographic marker should appear at the zero point at this gating level. The actual distance between the marker in the gated image and the zero point was measured and divided by the marker velocity, to calculate the time delay of the system. The beam on time delay was measured by examining the marker location in the first frame of each gated fluoroscopic playback. The final frame indicated the beam off time delay. Each gated movie produced 8 to 15 measurements of time delay for both beam on and off, depending on the period and exact length of recording. This process is shown graphically in Fig. 3. All gated measurements for imaging and treatment beams used gating levels of 50% amplitude or its phase equivalent.

We measured time delays on a Varian Acuity with RPM and two Varian linac kilovoltage onboard imagers (OBIs), (Varian Medical Systems, Inc., Palo Alto, CA). The systems examined had fluoroscopic frame rates of approximately 10 frames per second.

### B. Gated treatment

**Figure 4:**
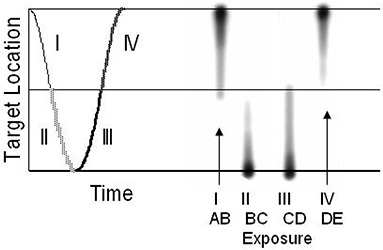
The method for finding time delay for the linear accelerator treatment beam. The curve at left represents the platform motion, and is separated into 4 regions (I is point A to B on Fig 3, II is B to C, III is C to D, IV is D to E). Gated exposures were obtained for each region. If there was no time delay, all streaks would be the same length, reaching from the top or bottom horizontal line to the middle horizontal line, depending on exposed region. Because A, C and E form stable points that do not move with changes in time delay, the difference in length of the exposed streak from this ideal indicated the beam on (for II and III) and beam off (I and IV) time delays. In all cases, the treatment beam responded late (i.e., beam‐on occurred after the IR markers had entered the gating region, and beam‐off occurred after they had left it).

To measure the time delay in the gated treatments, high sensitivity film (Kodak EDR‐2, Eastman Kodak Co, Rochester, NY)) was placed on the moving platform (Fig. 2) under a thin sheet of copper for buildup. A stationary 4 mm diameter cone, mounted just above the film, was used to obtain a small, well‐defined irradiated spot on the film. This small exposure produced a short streak on the moving film. The respiratory cycle was divided into 4 parts, as shown on Fig. 4. The film was exposed during part I of a single cycle using the gating system. The film was shifted, and an exposure obtained for part II, and so on. Four exposures were made for each measurement condition. The end‐inhale and end‐exhale positions (points A and C in Fig. 3, respectively; also the top and bottom horizontal lines in Fig. 4) are relatively stable using this setup, but a small time delay at mid‐cycle (points B and D in Fig. 3, respectively, also the middle horizontal line in Fig 4) results in a noticeable change in the streak length. The difference in exposure length, Δ*L*, from that expected from the known motion of the moving platform was converted to a time delay, *t*, using the known speed of the platform, *vp* (e.g., t=ΔL/vp) so a 2 mm change in streak length for a 5 sec period motion indicates a 0.095 sec time delay. We measured time delays for a number of different energy beams (6 MV, 10 MV and 15 MV), dose rates (300 and 600 MU/min), and periods of motion (3 to 6 sec), using 3 RPM systems on three linear accelerators (Varian 2100 C/D, 21EX and Trilogy). All systems used the same software configuration and version.

### C. Implications for patient treatment

**Figure 5:**
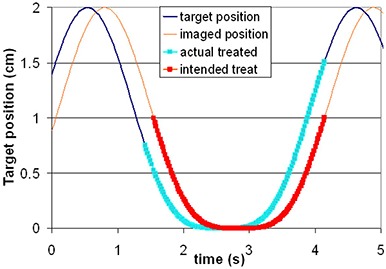
A model of the impact of time delay on delivered treatment. The apparent target position from the images (modeled as sine^4^, total motion=2cm,period=4sec) is shown as the light line, and is used to select a gating level, here 50%. Target motion is shown as thin lines. The actual target position at treatment is shifted from the imaged position by the sum of the time delays between the imaging and treatment systems. Our results indicate the treatment beam on is 0.13 sec after the image acquisition and beam off is 0.26 sec. Although one may locate a gated region based on the imaged position of the target (the thick red line), the actual target location treated is given by the thick blue line. Treatment delivery, therefore, begins after the target is within the gated region, decreasing the duty cycle. Treatment delivery stops after the target has left the gated region shown on the image, resulting in a geographic miss.

Treatment accuracy depends on the synchronicity of imaging and treatment time delays. Depending on the direction of the discrepancy, they may result in a decrease in the beam on time per cycle, reducing overall efficiency, or in geographic misses of the target. A graphical description of the impact of time delays on treatment is shown in Fig. 5. We model the motion of the target position as (sine^4^) function, with a total motion of 2 cm and a period of 4 sec, and gate based on amplitude at 50%. The phantom used for time delay measurement moved approximately in a sine wave, but any well‐known pattern of motion could be used to measure time delays. To assess the implications for patient treatments, we use (sine^4^) function. Higher order sinusoidal functions, though imperfect representations of irregular patient breathing patterns, have frequently been used to model patient breathing.^(^ ^7^ ^–^ ^12^ ^)^ Using the amplitude‐based gating time delays measured, the treatment beam on is 0.13 sec after the image was acquired, while the beam off is 0.26 sec. Treatment delivery, therefore, begins after the target is within the gated region, decreasing the duty cycle. Treatment delivery stops after the target has left the gated region shown on the image, resulting in a geographic miss.

**Figure 6:**
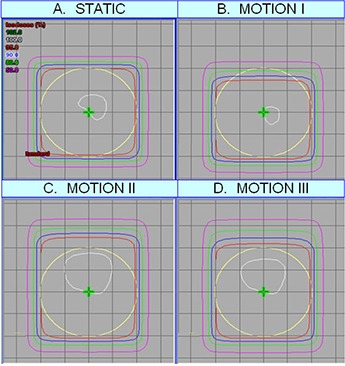
Effect of time delay error on isodoses. A 4 cm diameter circular target at a depth of 5 cm in a large, rectangular, homogeneous phantom is treated with a single 6 MV beam. The first plan (STATIC) is a simple treatment of a static target with a 5 cm by 5 cm square beam. In all other plans, we simulated target motion using a sine^4^ function with an amplitude of 2 cm, was amplitude gated to 1 cm motion from ‘end‐exhale’. In the second plan (MOTION I), we simulated a gated treatment with perfectly accurate gating (no time delay discrepancy). The third plan (MOTION II) shows the same gated treatment, but with a beam size increased by 1 cm larger beam in the direction of motion. This is the margin that is selected in some clinics, and is clearly overgenerous, increasing normal tissue dose. Finally, MOTION III demonstrated the effect of the non‐zero average time delay difference (0.26, measured for amplitude based gating) between imaging and treatment, as in Fig. 6. The over‐generous margin from MOTION II is clearly reduced by the asynchrony between the imaging and treatment time delays.

In order to examine a more clinical case, a number of treatments were simulated using the Eclipse (Varian Medical Systems, Inc. Palo Alto, California), as shown in Fig. 6. Each plan shows a 4 cm diameter circular target at a depth of 5 cm in a large, rectangular, homogeneous phantom. A single 6 MV beam is used to treat the target. The first plan (STATIC) shows the dose distribution delivered to a static target by a 5 cm by 5 cm square beam. For the other three plans, breathing motion was modeled using a (sine^4^) function with a total motion of 2 cm and a period of 4 seconds. The target motion was divided into 36 discrete steps. The effect of motion was simulated in the treatment planning system by applying a treatment beam centered at each calculated target location weighted by the target dwell time at that location. The dose from the beams was summed to gain an approximate dose distribution for a ‘moving’ target. In the second plan (MOTION I), an ideally gated treatment, with gating window (1 cm), was simulated. That is, only beams for which the target displacement from end‐exhale was less than or equal to 1 cm delivered dose. All other beams had zero weight. This plan demonstrated the effect of motion on dose distributions, which occurs in gated treatments with some residual motion. To compensate for this a margin is typically added. Therefore in the third plan, (MOTION II) we simulated another ideally gated treatment, this time increasing the beam size by 1 cm in the direction of motion only. In an ideally gated treatment, the target is always within the beam with this margin, but the normal tissue dose near the target is increased. In the final plan, we show the results of attempting to deliver the MOTION II plan with a non‐zero time delay difference. The actual time delays measured for amplitude‐based gating were used. When a time delay difference occurs, the treatment beam is turned on when the target is closer than 1 cm to the end‐exhale position but not turned off until it is about 1.5 cm from end‐exhale, as shown in Fig. 5.

**Figure 7:**
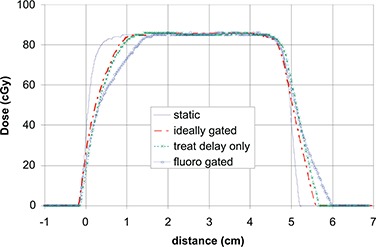
Profiles in the direction of motion of moving films. End‐exhale is at left. A profile on a static film with a 5cm×5cm6MV exposure is shown for reference. We selected gating levels by limiting apparent motion on fluoroscopy to 1 cm (phases 31 to 82 – ‘fluoro gated’ profile), which mimics the clinical situation. The ‘treat delay only’ profile was gated to phases 25 to 75%, which should correspond to 1 cm total motion if the treatment time delay is zero. This profile is effectively corrected for the imaging time delay. The ‘ideally gated’ profile is obtained by correcting the phase gating window for the treatment time delay.

Finally, we exposed a series of films with 5cm×5cm100MU,6MV beams at 600 MU/min at 1.5 cm depth, SAD. We exposed a static film, then three on the moving platform (sin motion, 2 cm total motion, 3 sec period). We determined the gating level from the clinical practice of measuring the total motion of a marker on fluoroscopy, and selecting phase based gating levels that showed only 1 cm total motion. The film was exposed using this gating level (31 to 82%). We made another exposure at the ideal gating level of 25 to 75%. Finally, we corrected for the treatment beam time delay on that unit (Linac 2 in Table 1) and gated at phase 22 to 73%. Profiles are shown in Fig. 7.

**Table 1:** The average time delay for motion period 3−5seconds for beam on/off, amplitude/phase based gating. Measurements are ± one standard deviation.

	*On‐Amp. (sec)*	*On‐Phase (sec)*	*Off‐Amp. (sec)*	*Off‐Phase(sec)*
Simulator	−0.04±0.06	−0.11±0.05	−0.19±0.08	−0.16±0.04
OBI 1	−0.04±0.03	−0.11±0.04	−0.17±0.06	−0.15±0.04
OBI 2	−0.03±0.05	−0.10±0.04	−0.18±0.09	−0.16±0.04
Linac 1	0.11±0.02	0.12±0.02	0.06±0.01	0.05±0.02
Linac 2	0.08±0.01	0.07±0.02	0.07±0.02	0.05±0.02
Linac 3	0.09±0.02	0.12±0.03	0.11±0.02	0.08±0.03

Note: Positive time indicates the beam is late turning on/off; negative indicates early turning on/off.

### D. Time delays and margins

One may derive a margin formula to estimate the effect of time delays. The time delay difference between the imaging and treatment systems may be plotted as a histogram, with an average, $t_{d}$, and standard deviation (if normally distributed), $\sigma_{t}$. The target velocity at gating will depend a number of factors, including gated window, and patient and tumor characteristics, but the average and standard deviation for the patient (if known) or population under study may be denoted $\nu_{d}$ and $\sigma_{v}$. Breathing patterns are, in general, non‐Gaussian;^(^ ^11^ ^,^ ^12^ ^)^ however, it is not yet known if the distribution of the target velocities at the gating level will be Gaussian or not. The average target motion, *m*, during a given time delay for a patient population may then be calculated as:

(1) $$m=\nu_{d}\times t_{d}$$

which will have a standard deviation, $\sigma_{m}$, of

(2) $$\sigma_{m}≅m\times {\left({\left(\frac{\sigma_{t}}{t_{d}}\right)}^{2}+{\left(\frac{\sigma_{v}}{v_{d}}\right)}^{2}\right)}^{1/2}$$

The target motion, *m*, will result in a random error described by $\sigma_{m}$, and, if $t_{d}$ is non‐zero, a systematic error as well. These errors occur only in the direction of motion, and so will increase the margin in that direction only. If the treatment goal is to have 100% of the target receive 95% of the dose, the impact of the systematic error may be calculated with an approximation based on duty cycle. Assuming a zero width penumbra and constant dose rate, the target must be within the treatment beam for at least 95% of the beam on time. In other words, if $t_{\text{avg}}$ is the average beam time per cycle, then the target should spend no more than 5% of $t_{\text{avg}}$ outside of the beam:

(3) $$5\%\;t_{avg}\;<\;|t_{d}|-{\;}^{M}{/}_{v_{d}}$$

where *M* is the margin in the direction of target motion. The sign of the difference in time delay will determine beam starts delivery when the target is outside the beam or does not turn off until after the target has moved out of the beam (as in the case measured here and noted in Fig. 5), but it is the magnitude of the discrepancy which determines *M* here. The total duty cycle is important to the margin selection, and larger duty cycles will reduce the impact of the time delays in the system. For a duty cycle of 2 sec (40% of a 5‐sec breathing cycle), a total time delay of <0.10sec will still maintain this limit. In reality, of course, a finite width penumbra will soften impact of time delays.

The random component of time delay errors will also have an effect on margins. Analogous to setup errors, these have both a random or day‐to‐day component (with standard deviation, σ), and preparation component (with standard deviation, Σ), which is systematic for a single course of a single patient. If a patient is imaged over a number, *N*, of breathing cycles at preparation, the preparation component can be correspondingly reduced. A typical goal may be that the minimum dose to the CTV be 95% for 90% of patients. There are a number of margin formulas in the literature, and applying van Herk's formula^(^ ^9^ ^,^ ^12^ ^)^ for example, the total margin required for time delay errors alone, $M_{\text{td}}$ will be

(4) $$\begin{array}{lll} M_{\text{td}} & \approx & v_{d}\times \left[t_{d}-\left(t_{ave}\times 5\%\right)\right]+2.5\Sigma +0.7\;ó\\ & \approx & v_{d}\times \left[t_{d}-\left(t_{ave}\times 5\%\right)\right]+2.5\sqrt{{\left({\;}^{\sigma_{m}}{/}_{\sqrt{N}}\right)}^{2}+\ldots }+0.7\sqrt{{\sigma_{m}}^{2}+\ldots } \end{array}$$

where the first term is a single known error with a non‐zero mean (which is without analogy in van Herk's work), the second term represents the preparation component of the errors, and the third is the random or day‐to‐day component. Uncertainties and systematic errors introduced by patient motion, coughing and changes to breathing pattern over time, as well as setup and other errors, are to be included as additional values in the second and third terms above.

## III. RESULTS

### A. Gated imaging

**Figure 8:**
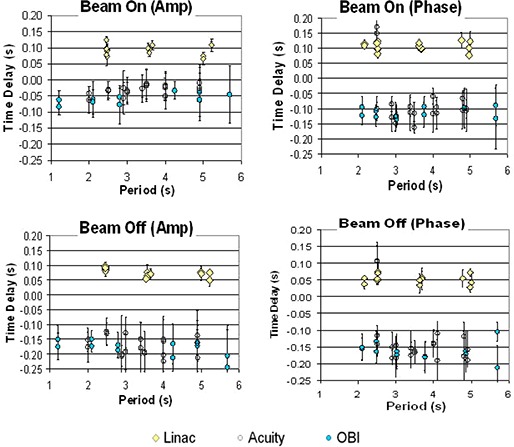
Time delays for three linear accelerators (3 energies, two dose rates are shown), one simulator, and two on‐board imagers for varying periods of motion and type of gating. The linear accelerators have a positive time delay indicating the beam on or off after the IR markers entered of left the gated region (late), while the imagers have a negative delay (early).

Table I shows the time delays measured under various conditions for three gated fluoroscopic imaging systems (one simulator and two kV OBI units). Figure 8 displays the period dependence of the same data. No trends in the time delay with period of motion were observed, and results were consistent between the two on‐board imagers and the simulator. The kV imaging gating was all performed retrospectively, and we found that the recorded image was, on average, obtained before the marker indicated it should be. This early imaging was consistent for beam on and off and for both phase and amplitude gating. Only a single measurement at short period for one OBI system demonstrated a case where the image was taken late. Accurate setup of the phantom was easy to verify by comparing the radiographic marker position at the cycle extremes.

### B. Gated treatment delivery

As shown in Table I and Fig. 8, the linear accelerator beam was turned on or off shortly after the markers indicated it should be, a time delay that is in the opposite direction to that of gated imaging. No period dependence trends in time delays were noted, although at very short periods of motion (below 3 sec) the system appeared to have difficulty in calculating the phase of motion, as demonstrated by the outliers in the treatment beam time delays. This meant the exposed film area was shorter than expected for the beam on at the high velocity position and longer for the beam off at that same position (see Fig. 4). Time delay measurements were consistent for the three energies measured and the three linear accelerators. Dose rate also did not have a significant effect on time delay measurement for the dose rates measured (400 vs 600 MU/min, data not shown). The variation in time delay was smaller for the treatment beams than for the imaging beams.

### C. Implications for patient treatment and margins

Figure 5 demonstrates the impact of time delay on delivered treatment. We model the motion of the target position as sine^4^, with a total motion of 2 cm and a period of 4 sec, and gate based on amplitude at 50%. In this case, treatment delivery, therefore, begins after the target already 0.75 cm is within the gated region, and does not stop until the target is over 1 cm outside of the gated region. The first effect will decrease the duty cycle and increase the normal tissue dose, while the second means that the target will be missed during part of the treatment. The clinical impact of this will depend on a number of factors. For instance, this effect will be less important for larger penumbra beams, and will decrease with increasing duty cycle, and patient breathing period. More mobile tumors and tumors moving rapidly at the gating levels will be more likely to be impacted.

Given the variability in patient breathing patterns, it is difficult to assess thoroughly the impact of time delay discrepancies, but an increase in the ITV margin may be needed compensate for the time delay between imaging and treatment gating. To translate these time errors into distances in order to assess their effect on patients, one must multiply by the tumor velocity at the gated level, (Eq. (3)). Information on average tumor speeds in the literature is sparse, but Shirato et al.^(13)^ measured internal fiducial marker motion in peripheral lung tumors in 21 patients. Gating levels are typically chosen at high fiducial velocity points in the middle of the respiratory cycle (rather than at the relatively stationary end‐exhale or end‐inhale), and this group reported maximum average speeds of 8.3 to 72.6 mm/sec (average 21.1±18.9mm/sec,1SD), and a median average speed of 9.9±5.4mm/sec. Again, using the measured difference in time delay between imaging and treatment beams, (amplitude, beam off=0.26±0.08sec) and the median velocity and its standard deviation from Shirato et al., and assuming an average beam on time per cycle $\left(t_{\text{avg}}\right)$ of 2 sec and imaging over 5 cycles (N=5) in Eq. (4), the increase in the ITV margin *from time delay alone* must be 4.5 mm in the direction of the target motion. More often, gating levels are chosen near mid‐cycle, which corresponds with the maximum of the target velocity. When the target velocity nears the maximum, ITV increases of up to 13 mm are necessary, simply to account for the differences in time delays. A large portion of this margin results from the last term in Eq. (4), which accounts for the variation in velocity. This may be reduced with improved knowledge of patient target motion. Margins to account for hysteresis in the breathing motion, phase difference between external surrogates and internal target motion, and changes in the breathing pattern over treatment must be included over and above this term.

Figure 6 simulates how motion and the systematic component of the time delay errors (the first term in Eq. (4)) may impact target dose. A treatment, gated under ideal conditions (no time delay), to allow 1 cm of motion (50% amplitude) is shown, and requires an increased margin (MOTION I) compared to the static case. A 1 cm margin in the direction of motion, for a gated treatment allowing 1 cm motion, is typical of the margin selection currently in our centre (MOTION II). This is, in fact, more than is necessary to cover the moving CTV, since the CTV is at end‐exhale for most of the beam on time, well away from the gated position. This conservative approach to margin selection will increase dose to surrounding normal tissue. The final simulation (MOTION III) includes the systematic component of the time delay errors (the first term only in Eq. (4)), and coverage in the direction of motion is noticeably impacted. In this case, however, the motion margin is generous enough to maintain target coverage. If a more analytic approach to margin determination for moving targets were implemented, time delay errors must be included.

Figure 7 demonstrates that time delays do result in measurable changes in the treatment of moving targets. This measurement was performed on the linac with the smallest imaging and treatment time delays. However, the degradation in the delivered profiles is clearly demonstrated.

## IV. DISCUSSION

Commissioning of gating systems should include an analysis of both the time delay and the dosimetric characteristics of the gated delivery for each linear accelerator used. The latter include dose linearity, fatness and symmetry for the range of doses, dose rates, and gating frequencies to be used clinically. Overall, linear accelerators appear very accurate at delivering dose in gated modes, for static as well as dynamic deliveries.^(^ ^8^ ^,^ ^14^ ^–^ ^18^ ^)^ Our results are consistent with these findings. A few articles have addressed the measurement of time delays. Jin and Yin^(6)^ measured the time delay for the ExacTracGating system (beta version) using a series of port‐films on which the image was exposed for a 6% window at various points in the cycle. They measured the position of moving infrared markers and, from this, calculated an average time delay of 0.17±0.03sec. Tenn et al.^(3)^ measured the delay between the time the gating signal is sent to the linear accelerator and the time the beam is initiated (that is, *the linear accelerator latency period*) to be 60±20ms. This linear accelerator latency period, often quoted by manufacturers, forms a component of the overall time delay, but margin calculations must be based on the overall time delay, which includes delay in the detection of motion by external markers and analysis by the gated system, in addition to linear accelerator latency. The article did identify an error related to the exposure time, but no time delay values for the kV imaging system were quoted. We expect time delays measured on different gating systems, software versions, and with different imaging systems and/or linear accelerator to be different from those measured here due to their unique predictive filters, motion analysis, latency periods and so on.

The selection of the optimum gating window may be based on gated CT images, and the accuracy of gated image acquisition for one system is discussed by Guana.^(4)^ Guana found an overall delay of 1.75 sec for the first axial scan and 0.75 sec for subsequent scans. That group divided the overall time delay into the triggering delay (250 ms), scanning delay (half the scan time=0.5sec for a 1 sec scan) and a mechanical start‐up delay (1 sec) for the first slice taken when the gantry starts to spin. The time delay measured in our experiment corresponds to the overall time delay as reported by Guana. While the RPM system investigated allowed the user to enter these delays, the system did not compensate for them. Time delay exists for the RPM camera and hardware/software to capture and process the respiratory motion signals and generate a CT trigger signal. The manufacturer quotes this as between a few ms^(4)^ and 50 ms (personal communication), with no distinction between the time delay for starting and stopping the linear accelerator beam. The frame time of the infrared camera is 33−40ms.^(4)^ Our studies of scan delay in gated fluoroscopic imaging show significantly smaller time delays than for 4DCT.^(4)^ Clinics interested in implementing 4DCT for planning gated treatments will need to take the larger time delays into account.

In our system, the imaging gating system retrospectively gated the fluoroscopic images. Our measurements show each image was in fact recorded before the IR markers were at the indicated phase or amplitude. Fluoroscopic images were obtained at a rate of approximately 10 frames per second, under clinical conditions. This low sampling frequency led to a higher variability in the time delay measurement for imaging compared to treatment. Viewing a gated patient image set over several breathing cycles will help to reduce this uncertainty.

One might expect the imaging systems, which gate retrospectively, to have a smaller time delay than the prospectively gated treatment systems. Our measurements show that this was not always the case. The regular motion of our phantom may represent a best‐case scenario for the predictive power of the gated treatment system while, in theory, the retrospectively gated systems should be less affected by the regularity of motion. A more clinical variable cycle breathing phantom must be developed to test such systems more completely. In contrast, the treatment system was prospectively gated. There are two components of a prospectively gated system: the software delay and the linear accelerator latency period. Different linear accelerators using the same RPM system may still have a different time delay because the linear accelerator latency period might be different. Similarly, the BrainLab gating system on a Varian linear accelerator, for example, also has a different time delay because of the differences in the software's predictive filter.

We have tabulated the results for phase‐ and amplitude‐based gating separately. One may anticipate that the predictive filters for these two types of gating may employ different mechanisms, although the commercial software involved operates as a black box. The response rate for prospectively gated treatment beams may demonstrate differences between gating types. Calculations of maximum inhale points for phase‐based gating, and maximum and minimum amplitudes, may also affect the time delays. However, with this software version, no significant differences between phase‐ and amplitude‐based gating were detected. Our phantom had regular motion with a constant amplitude and period, and significant differences between phase‐ and amplitude‐based gating time delays may become apparent under more clinical conditions.

A growing number of investigators are examining the way margins and tolerance limits for radiotherapy equipment are set, moving beyond the simple adding in quadrature^(^ ^19^ ^,^ ^20^ ^)^ to methods based on the way real errors compound in radiotherapy systems with the results directly related to the accuracy and precision of delivered patient treatments.^(^ ^21^ ^–^ ^24^ ^)^ The time delay of imaging and treatment gating will combine linearly if they are asynchronous. Time delay differences resulting in delayed delivery, where the target is within the treatment region such as late treatment beam on or early treatment beam off compared to imaging beams, will decrease the beam on time per cycle, reducing overall efficiency. These differences introduce a systematic error with does not ‘blur out’ during the multiple breathing cycles. Limits on these delays should be set based on achievability and, if relevant, radiobiological considerations. Other time delays, such as early treatment beam on and late treatment beam off compared to imaging, may result in geographic misses of the target. Limits set on these time delays of the system should be calculated based on the desired margin or the margin should be selected based on these delays. Ideally, this should be tied into an overall assessment of tumor control probability and normal tissue complication rate and, ultimately, to patient survival.

However, tolerances and limits within radiotherapy systems are currently selected based largely on clinical experience and achievability, without the corresponding theoretical basis. For our system, treatment beam time delay (0.08 sec: the treatment beam begins and ends delivery after the IR target enters or leaves the gated region) adds linearly to the imaging time delay (e.g. −0.12sec: the images are shown at time points earlier the time at which they were actually obtained). The average total delay is 0.20 sec. This type of error will result in a systematic misalignment between gated imaging and treatment beams (Fig. 5) as well as increased residual motion and decreased efficiency. The patient impact is seen directly in the required ITV margins in the direction of tumor motion at the gating level. These margins are strongly affected by the tumor velocities. Increasing tumor velocity increases both the systematic and random components of error, while variability in patient breathing patterns will alter the random components. Fast moving tumors move outside the radiation beam more quickly. Actual tumor velocities at gated cutoffs will depend on gating level, tumor type and location, and patient respiration, among other factors. Choosing a margin to completely cover the moving CTV is one conservative approach, but can result in increased dose to surrounding normal tissues. More aggressive margin selection depends on detailed knowledge of target motion.^(25)^ To reduce such margins, gating at the maximum velocity may not always be the best option. Also, the use of patient‐specific data can greatly reduce the velocity variability, thus minimizing the necessary margin. Increasing the duty cycle makes time delays less important and reduces the margin necessary to compensate for them. The margin sizes calculated to cover the time delays found in this work apply only to the systems and versions that we investigated. Each center should measure the time delay for each system used, calculating its impact on required margins.

Margins to account for time delay form only one part of the margins required for moving targets. Setup errors for a moving target, residual motion in the gated window, changes in respiration, target correlation with external markers – these are among the many other factors which will determine the total margin size. How motion margins should combine with each other and margins for other errors remains a topic of debate.^(^ ^21^ ^,^ ^24^ ^,^ ^26^ ^)^ Our simple simulation of the effect of the systematic error in time delay in our system demonstrated a shift of several millimeters in the isodose lines under reasonable motion conditions.

This paper, and the others we are aware of that calculated time delays, are based on the motion of phantoms having constant periods and amplitudes. In patients, however, both the frequency and amplitude of respiration are quite variable. To investigate the system's response to this variability would require custom‐built programmable motion phantoms. This remains an area open to further exploration.

## V. CONCLUSIONS

The imaging systems examined here have average (beam on and off both amplitude and phase) timer delay of −0.12sec, while the average timer delay for the treatment delivery system is 0.08 sec. The sum of the total delay is 0.20 sec, despite the fact that the individual delays are smaller. This type of error will result in a potentially significant systematic error in treatment as residual motion is increased and efficiency decreased. Conversely, if the time delays are in the same direction for both systems, they will cancel each other out. We therefore recommend that tolerances for time delays in gated imaging and treatment be set together rather than individually. The time delay values found in this manuscript apply only to the system and version investigated, but we have presented a simple method for determining the time delay that can be applied to any system. We demonstrated a method of calculating the increase in required margins due to the systematic and random components of time delay. A simulation of the impact of systematic error showed it can cause significant changes in dose distribution delivered to a moving target under clinical conditions.

## ACKNOWLEDGEMENTS

The authors thank Dr. Peter Dunscombe, Shannah Murland, and Dr. Harold Lau for their valuable insights.
